# An Update on Pharmacological Potential of Boswellic Acids against Chronic Diseases

**DOI:** 10.3390/ijms20174101

**Published:** 2019-08-22

**Authors:** Nand Kishor Roy, Dey Parama, Kishore Banik, Devivasha Bordoloi, Amrita Khwairakpam Devi, Krishan Kumar Thakur, Ganesan Padmavathi, Mehdi Shakibaei, Lu Fan, Gautam Sethi, Ajaikumar B. Kunnumakkara

**Affiliations:** 1 Cancer Biology Laboratory and DBT-AIST International Centre for Translational and Environmental Research(DAICENTER), Department of Biosciences and Bioengineering, Indian Institute of Technology Guwahati, Assam 781039, India; 2 Musculoskeletal Research Group and Tumour Biology, Chair of Vegetative Anatomy, Institute of Anatomy, Ludwig-Maximilian-University, 80336 Munich, Germany; 3 Department of Pharmacology, Yong Loo Lin School of Medicine, National University of Singapore, Singapore 117600, Singapore

**Keywords:** boswellic acid, chronic diseases, molecular targets, pharmacokinetics, bioavailability

## Abstract

Natural compounds, in recent years, have attracted significant attention for their use in the prevention and treatment of diverse chronic diseases as they are devoid of major toxicities. Boswellic acid (BA), a series of pentacyclic triterpene molecules, is isolated from the gum resin of *Boswellia serrata* and *Boswellia carteri*. It proved to be one such agent that has exhibited efficacy against various chronic diseases like arthritis, diabetes, asthma, cancer, inflammatory bowel disease, Parkinson’s disease, Alzheimer’s, etc. The molecular targets attributed to its wide range of biological activities include transcription factors, kinases, enzymes, receptors, growth factors, etc. The present review is an attempt to demonstrate the diverse pharmacological uses of BA, along with its underlying molecular mechanism of action against different ailments. Further, this review also discusses the roadblocks associated with the pharmacokinetics and bioavailability of this promising compound and strategies to overcome those limitations for developing it as an effective drug for the clinical management of chronic diseases.

## 1. Introduction

Chronic disease can be defined as a physical or psychological state that leads to functional limitations or requires constant observation or treatment for a long period. Worldwide, chronic diseases have hampered the health and living conditions of many [1]. Many of the universally used clinical drugs (especially the biologics) these days bear the shortcomings of side effects and high treatment cost [2]. Thus, numerous natural compounds, which have identified as potent modulators of signaling and epigenetic pathways leading to cancer, are under development presently [3]. Natural products have gained considerable attention as they are plentiful sources of diverse compounds, which can function as biologically active drugs against different chronic diseases [4,5,6,7,8,9,10,11,12,13,14]. These plant-derived molecules have significantly enhanced the existing medicinal system. For example, in a developing nation like India, around 65% of the country’s population gets benefitted by the use of phytomedicines that play an essential role in the health management system. satisfying. In developed nations like the USA, the sale of phytomedicines has registered a sharp incline in recent years. Around 80% of the African population relies on the use of phytomedicines to meet their health care needs through the use of traditional medicines. According to the WHO, nearly 80% of the world’s population uses phytomedicines for the management of various ailments [15,16,17,18,19,20,21,22,23,24].

For generations, numerous natural compounds extracted from different plants and showing significant pharmacological properties, have used for treating various chronic diseases. To date, about 10,000 phytochemicals comprising of tannins, flavones, triterpenoids, steroids, saponins, and alkaloids have identified, and many more are yet to discover. It is believed that the antioxidant activity of phytochemicals increases their action synergistically, as numerous reports evidenced that overproduction of oxidants (reactive oxygen species and reactive nitrogen species) causes many chronic diseases such as cardiovascular diseases (CVD), diabetes, and cancers [25,26,27,28,29,30,31].

Boswellic acid (BA) is one such phytochemical, obtained from the gum resin of the *Boswellia* species, that possibly aid in the treatment of different chronic diseases. Traditionally, the gum resins of *Boswellia* species found its applications in various adhesives, cosmetic preparations, coating materials, the incense used in cultural rites and rituals, and many more. It is one of the most essential and commonly used components in conventional Ayurvedic and Unani medicines, which have proven to be extremely effective in relieving numerous inflammatory, gastrointestinal, hormonal, and microbial diseases [32]. The conventional drug is said to have the properties of an anti-inflammatory, antiseptic, expectorant, anxiolytic, antineurotic, analgesic, and tranquilizing drug [33]. Various preclinical and clinical studies have established that it exhibits substantial potential in the management of inflammatory ailments such as asthma, arthritis, cerebral edema, chronic bowel diseases, chronic pain syndrome, cancer, etc [34,35].

### 1.1. Sources and Chemical Analogues of Boswellic Acid

**Figure 1:**
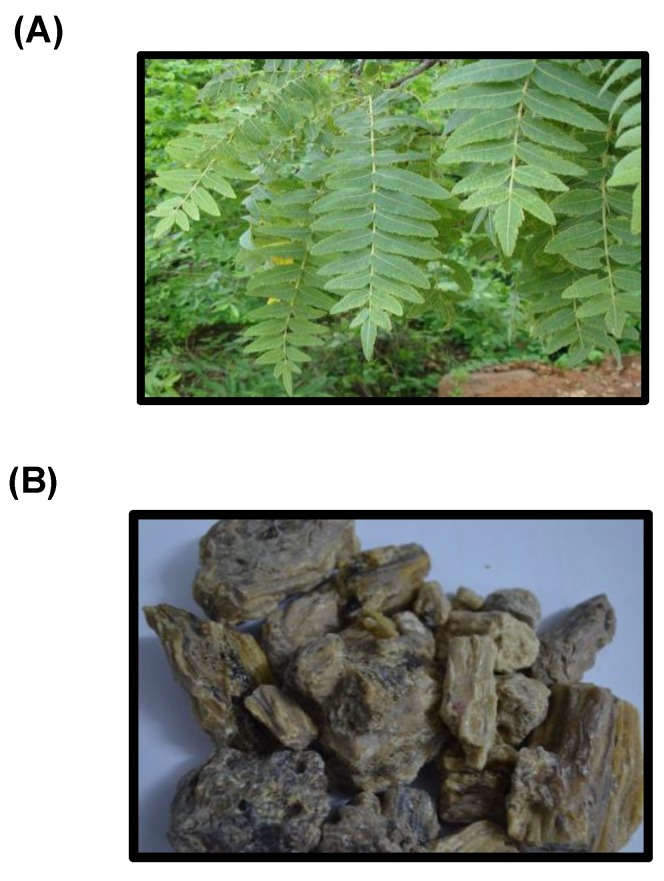
(**A**) Boswellia (Pankaj Oudhia/www.discoverlife.org) and (**B**) Boswellia gum resin.

BA comprises of a series of pentacyclic triterpene molecules, generated by the trees in the genus *Boswellia*, usually known as Indian olibanum, salai guggal, loban, or kundur, and is found to be effective against many diseases. Categorized under the Burseraceae family, these are moderate to large-sized branching trees prevailing over the mountainous regions of India, Northern Africa, and the Middle East. The genus *Boswellia* consists of roughly 25 species widely dispersed in Arabia, the Northeastern coast of Africa, and India [32,36] (Figure 1).

**Figure 2:**
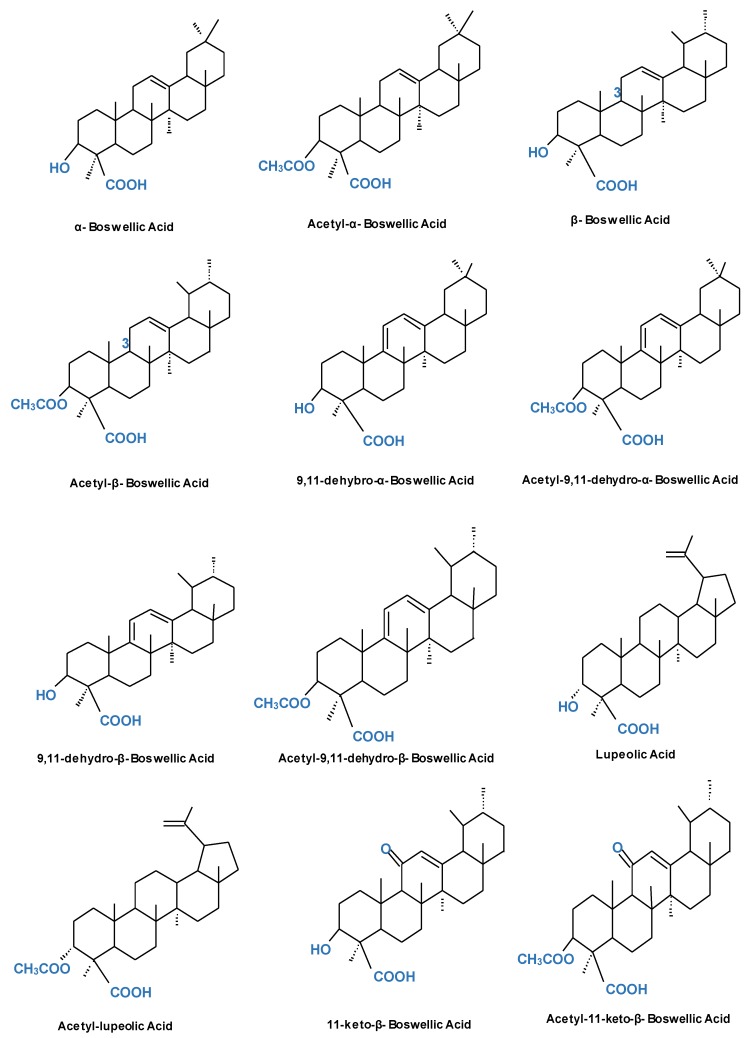
Structure of different triterpenic acids of the Boswellia species.

In India, *Boswellia* is mostly found in Andhra Pradesh, Gujarat, Madhya Pradesh, Jharkhand, and Chattisgarh. These are the most viable sources of *Boswellia*. The gum resin of *B. serrata* and *B. carteri* contain as many as 12 different types of BAs, but among these the six major acids identified are α and β-boswellic acids (BA), acetylated α and β-boswellic acids (ABA), 11-keto-β-boswellic acid (KBA), and 3-O-acetyl-11-keto-β-boswellic acid (AKBA), which are liable for inhibiting the enzymes involved in inflammation. Several added BAs extracted from *Boswellia* are 9,11-dehydro-α-BA and 9,11-dehydro-β-BA, and their respective acetylated forms acetyl-9,11-dehydro-α-BA and acetyl-9,11-dehydro-β-BA. Some additional chemical components of *Boswellia* include lupeolic acid and acetyl-lupeolic acid, incensole acetate, incensole oxide, and isoincensole oxide. Studies have also described the incidence of a pentacyclic triterpenediol combination of 3α,24-dihydroxyurs-12-ene and 3α,24-dihydroxyolean-12-ene, serratol, α-thujene, tirucall-8,24-dien-21-oic acids, oilbanumols D-G, α-pinene, and octyl acetate in the crude *Boswellia* gum resin extract. However, KBA and AKBA have proven to be the most potent in downregulating the production of cytokines and inhibiting the enzymes responsible for inflammatory responses. Hence, these have reported as efficient therapeutics against different chronic diseases [32,37,38,39,40,41], (Figure 2).

### 1.2. Pharmacological Activities of Boswellic Acid

The pharmacological activities of BA are attributed to its aptness to induce anti-inflammatory, expectorant, antiseptic, anxiolytic, anti-neurotic, analgesic, tranquilizing, and antibacterial effects [33]. It can modulate diverse targets such as enzymes, growth factors, kinases, and transcription factors, as well as receptors, which allow it to stimulate apoptosis, cell cycle arrest, etc. [36]. It can also inhibit different signaling pathways [42] related to cell survival [43], proliferation [44], and metastasis [45].

## 2. Molecular Targets of Boswellic Acids

**Figure 3:**
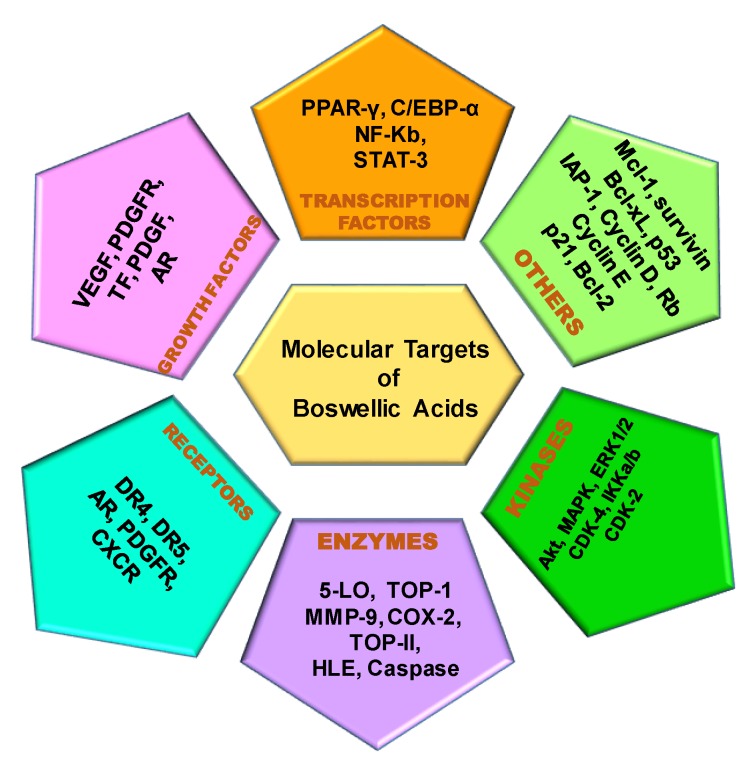
Molecular targets of Boswellic Acids and their analogues.

Different chronic diseases, including CVDs, diabetes, and cancers, arise from the alteration of multiple signal transduction cascades and can affect people of all ages [46,47]. It is now well established that BA is a multitargeting agent. It can modulate several molecular targets, including enzymes, growth factors, kinases, transcription factors, receptors, and others related to the survival and proliferation of cells [36], (Figure 3). Increasing lines of evidence indicate that nuclear-factor kappaB (NF-κB) and signal transducer and activator of transcription 3 (STAT3) activation can lead to survival, angiogenesis, and metastasis of the cancer cells [43,48,49,50,51,52,53,54,55,56,57,58,59,60,61,62,63,64,65,66,67,68,69,70,71,72,73,74,75,76,77,78]. Hence, studies aimed at targeting these pathways may pave the way for both the prevention and treatment of cancer and other chronic diseases [79]. In the year 2006, Poeckel and Werz reviewed the molecular mechanisms essential for the biological activities of BAs, where they have discussed its target molecular mediators, such as 5-lipoxygenase, human leukocyte elastase, topoisomerase I, II, and IκB kinases. Furthermore, BAs were reported to have the ability to differentially-regulate the Ca^(2+/−)^ and mitogen-activated protein kinases (MAPK) signaling cascades in blood cells, and also affect the functional cellular processes that are imperative for inflammatory reactions and tumor growth [80,81,82]. Alterations of these inflammatory pathways can lead to serious diseases including ulcerative colitis, rheumatoid arthritis, bronchial asthma, chronic colitis, Crohn’s disease, peritumoral brains edemas, etc., and BAs are known to target them through the above mentioned molecular mediators [83]. Several analogues of BA were also reported to target the key mediators involved in the pathogenesis of cancer including NF-κB, STAT3, peroxisome proliferator-activated receptor gamma (PPAR-γ), CCAAT enhancer-binding proteins alpha (C/EBP-α), cyclooxygenase-2COX-2, matrix metallopeptidase 9 (MMP-9), Caspase, Cyclin D, Cyclin E, p21, p53, Rb, Bcl-2, Bcl-xL,Mcl-1,inhibitor of apoptosis (IAP-1), survivin, vascular endothelial growth factor (VEGF), platelet-derived growth factor (PDGF), androgen receptor (AR), death receptor 5 (DR-5), CXCR4, PDGFR, Akt, ERK1/2, p38 MAPK, cyclin-dependent kinase (CDK) -2, CDK-4. These mediators are involved in different processes of cancer development [84,85,86], such as uncontrolled proliferation [87], unresponsiveness to inhibitory signals, resistance to apoptosis [88], angiogenesis [89], metastasis [90,91,92,93,94,95,96,97,98,99,100,101]. Among these molecular targets, NF-κB and Akt play an important role in cancer progression regulating cancer cell proliferation, survival, invasion, metastasis, and high mortality of patients [77,78]. Moreover, they are also responsible for inducing chemo and radioresistance in the cancer cells [102,103].

## 3. Potential Role of BAs in the Treatment of Chronic Diseases

**Figure 4:**
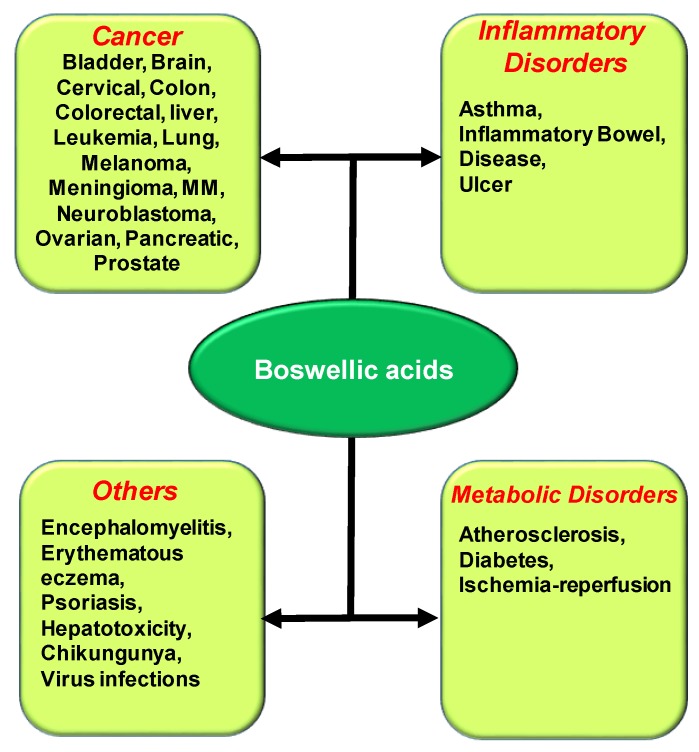
Biological activities of Boswellic Acids against diverse chronic diseases.

As aforementioned, BA is a multitargeted compound, which enables its use against diverse diseases (Figure 4). The prospective of BAs in managing various chronic diseases is well evidenced by a number of preclinical studies through their ability to modulate multiple mediators involved in the pathogenesis of diverse diseases (Table 1).

### 3.1. Arthritis

Arthritis predominantly arises due to inflammation of joints and the connective tissues surrounding them. Osteoarthritis, being the predominant of all forms, affects a wide range of the population all over the world [104]. A study on the effect of BAs in bovine serum albumin (BSA)-induced arthritis reported that on oral administration, BAs (25, 50, and 100 mg/kg/day) noticeably mitigated the leucocyte population and inhibited its infiltration into the knee joint as well as the pleural cavity in a BSA-injected knee. Also, the electrophoretic pattern of the proteins present in the synovial fluid was altered [105]. Additionally, when BA was conjugated with an active metabolite rhein and administered at the specified dose level of 15.73 mg/kg, p.o. (BID), it reduced the diameter of the knee and normalized the biochemical and hematological anomalies in rat models of collagenase-induced osteoarthritis [106]. Another study demonstrated that under topical treatment, the concentrations of BA in synovial fluid increased two- to six-fold as compared to its level in plasma. Loss of cartilage in mice was found to reduce considerably after oral or topical treatment with BAs compared to vehicle control [107].

### 3.2. Asthma

Asthma is rising as a severe global health issue, which is characterized by airway hyperresponsiveness, airway inflammation, enhanced mucus production, airway epithelial wall shedding, and an increase in the IgE levels. An investigation on the anti-asthmatic potential of BA in a murine model of asthma reported suppression of allergic airway inflammation, AHR, OVA-specific IgE, and Th2 cytokines secretion were in treated groups. Furthermore, the expression of p-STAT6 and GATA3 were also suppressed in a dose-dependent manner [110]. In another in vivo study, the effect of BA was analyzed by injecting a sensitization liquid (0.15 mL aluminum hydroxide gel at 88.67 mg/mL and 0.05 mg ovalbumin) intraperitoneally in an asthma model, and it was found to minimize the symptoms by abrogating p-STAT6 followed by a reduction in GATA3 expression [111].

### 3.3. Atherosclerosis

Atherosclerosis occurs due to the formation of plaque inside the blood vessels leading to thickening of the arteries. An investigation on the effect of AKBA in apolipoprotein E-deficient (ApoE−/−) mice showed that it inhibited NF-κB, a vital element for the development and prognosis of various inflammatory diseases. Thus, this finding suggests that the plant resins from the *Boswellia* family can provide a substitute for conventional treatment strategies for chronic inflammatory diseases like atherosclerosis [86].

### 3.4. Cancer

Cancer is one of the most fatal diseases of mankind, with extremely high incidence and mortality rate. In the year 2012, it was estimated that about 14.1 million people suffered from the disease and 8.2 million people succumbed to death, whereas in the year 2018, the number of deaths increased to 9.8 million worldwide [162,163,164,165,166]. Notably, the majority of the existing drugs exert severe side effects and are mostly ineffective due to the development of chemoresistance [23,167,168,169]. This has led to shifting of attention towards natural products such as butein, emodin, curcumin, epigallocatechin gallate (EGCG), celastrol, honokiol, resveratrol, etc. which have shown high potential against various types of cancer [23,170,171,172,173]. Moreover, different studies have shown the efficacy of BA in the prevention and treatment of breast, bladder, cervical, prostate, colorectal, head and neck, liver, lung, and pancreatic cancers, etc. [36].

#### 3.4.1. Breast Cancer

In order to explore the potential of 3-O-Acetyl-β-BA (3-OAβBA) and *B.serrata* extract (BSE) in the prognosis and treatment of breast cancer, an in vitro study was performed on MDA-MB-231 cells. Both BSE and 3-OAβBA were found to be effective against triple-negative breast cancer by upregulating the expression of PERK-ER/UPR (protein kinase RNA-like endoplasmic reticulum kinase-endoplasmic reticulum/unfolded protein response) pathways that can regulate activated programmed cell death (APCD). Also, BSE and/or 3-OAβBA considerably downregulated the expression of oncogenes (OG) and upregulated the expression of tumor suppressor genes (TSGs), which includes glutathione-depleting ChaC glutathione-specific gamma-glutamylcyclotransferase 1 (CHAC1) and the mTOR inhibitors–sestrin 2 (SESN2) Tribbles homolog 3 (TRIB3), homocysteine-inducible, endoplasmic reticulum stress-inducible, ubiquitin-like domain member 1 (HERPUD1), and cystathionine gamma-lyase (CTH) [112].

#### 3.4.2. Bladder Cancer

Nearly, 430,000 people are diagnosed with bladder cancer annually, and 165,000 people die every year all across the globe [174]. To evaluate the anti-cancer effect of frankincense oil, (main component of which is BA), an in vitro study on J82 (human bladder cancer) and UROtsa cells (immortalized normal bladder urothelial cells) was performed. Treatment with frankincense oil exerted cytotoxic effects on the J82 cell line but had minimal effect on UROtsa cells. Thus, frankincense oil was found to differentiate between cancerous cells and normal cells and caused the suppression of tumor cell viability [113].

#### 3.4.3. Brain Cancer

Approximately 256,213 individuals, which includes 116,605 females and 139,608 males, in the year 2012, were diagnosed with a primary malignant brain tumor, globally [175]. Glaser et al*.,* in 1999, observed that at low micromolar concentrations, BAs showed cytotoxicity against malignant glioma cells [122]. Further, the pure extract of the gum resin of *B.serrata* and various analogues of BA including AKBA, BBA, and cyano enone of methyl boswellates (CEMB) have shown cytostatic and apoptosis-inducing activity against glioma cells [124,125].. Studies by Park et al*.,* on meningioma cells also suggested that the cytotoxic action of AKBA might, at least in part, be mediated by Erk signal transduction pathway inhibition. Furthermore, in vivo studies on an immunocompromised mice model (C6 glioma tumor xenograft) reported that intratumor administration of CEMB significantly inhibited the tumor growth, signifying the potent antitumor effect of CEMB [123].

#### 3.4.4. Cervical Cancer

One of the most common reasons for female malignancy in the world is cervical cancer, causing approximately 265,700 deaths annually [176]. In cervical cancer, treatment with 3-α-propionyloxy-β-BA (POBA) caused PARP cleavage, which consequently led to a cell cycle arrest, DNA fragmentation, and loss of mitochondrial membrane potential in SiHa cells [115].

#### 3.4.5. Colon Cancer

The apoptotic and antiproliferative effects of the analogues of BA, such as BBA, KBA, and AKBA in colon cancer cells, were analyzed. It was observed that AKBA could induce apoptosis through caspase activation and the p21-dependent pathway [87,114]. Moreover, studies on APC^(Min/+)^ mice have shown the chemopreventive action of AKBA against intestinal adenomatous polyposis by inhibiting Wnt/β-catenin and NF-κB/Cox-2 signaling pathways [91]. In vitro studies on human colon cancer cells further showed that the potent anticancer effects of BA might be mediated via induction of apoptosis and cell cycle arrest, as well as abrogation ofPI3K/Akt signaling pathway [42].

Also, AKBA affected the growth of colorectal cancer cells through genetic (Ki-67 and CD31) and epigenetic modulations (demethylation and miRNA regulation) [34,116,117]. Furthermore, AKBA, in combination with curcumin, showed antitumorigenic effects in vitro and in vivo by regulating specific cancer-related miRNAs such as miR-34a and miR-27a in colorectal cancer cells [177].

#### 3.4.6. Leukemia

The antitumor activity of BA and its analogues, such as BBA, KBA, AKBA, and PKBA, were studied in different leukemic cell lines such as HL-60, K562, MOLT-4, THP-1**,** CCRF-CEM, ML-1, NB4, SKNO-1, and U937 cells. Results showed that the treatment with BA exerted cytostatic and cytotoxic effects through the induction of apoptosis. Upon examining the molecular mechanisms involved, it was found that the treatment resulted in the attenuation of topoisomerases I and II, the release of cytochrome c, the loss of mitochondrial membrane potential, activation of caspases, and cleavage of PARP. It was also reported that the treatment led to the decreased expression of MMP-1, MMP-2, and MMP-9 mRNAs; along with the secretions of TNF-α and IL-1β; reduced the phosphorylation of ERK1/2, p38 MAPKs; and disrupted PI3K/AKT/Hsp-90 cascade [95,97,126,127,128].

#### 3.4.7. Liver Cancer

Around 782,500 new cases and 745,500 cancer-related deaths have occurred due to liver cancer or hepatocellular cancer in the year 2012 [66,178,179,180,181,182,183,184,185]. When the effects of KBA and AKBA were evaluated, they were found to inhibit proliferation and induce apoptosis through the caspase-8-dependent pathway in liver cancer cells [130]. Also, BSE, when administered as monotherapy and in combination therapy with DOX, caused an augmentation in caspase-3 activity, TNF-α, and IL-6 levels, thus showing growth-modulatory and apoptotic actions in hepatocellular carcinoma cells [186].

#### 3.4.8. Lung Cancer

An in vitro study on H446 cells was performed to explore the antitumor potential of 11-carbonyl- BBA. It was found to activate JNK signaling pathway, cause the cleavage of PARP, and downregulate survivin protein expression, thus showing inhibitory effects on lung cancer cells [131]. Moreover, a study focusing on the potential of POBA showed that POBA initiated PARP cleavage on HOP-62 lung cancer cells. As a consequence of the treatment, induction of apoptosis, as well as cell cycle arrest occurred in lung cancer cells [115].

#### 3.4.9. Prostate Cancer

GLOBOCAN 2012 reported that prostate cancer accounts for nearly 1.1 million new cases all across the world [162,187,188,189,190,191,192]. AKBA was shown to elicit cell death and reduce cell proliferation in PC-3 prostate cancer cell lines by abrogation of the activated NF-κB signaling pathway via interception of IκB kinase activity and activation of caspase-3 [88,113]. In LnCaP and PC-3 prostate cancer cells, AKBA showed apoptotic effects driven by the death receptor 5-mediated pathway. Besides that, caspase-3 and caspase-8 activation, as well as PARP cleavage induction, were evidenced [99]. In another study, AKBA was found responsible for the suppression of VEGFR2-mediated angiogenesis in prostate cancer [101]. Studies by Liu et al., showed that AKBA suppressed docetaxel-resistant prostate cancer cells via blockage of STAT3 and Akt signaling pathways [138]. A semi-synthetic triterpenoid derivative, 3-cinnamoyl-11-keto-beta-BA (C-KβBA), demonstrated specific antiproliferative and proapoptotic effects in cancer cell lines such as PC-3, LnCaP, and DU-145, as well as in PC-3 prostate cancer xenografts, by downregulating the activation of p70 ribosomal S6 kinase [122].

#### 3.4.10. Pancreatic Cancer

Pancreatic cancer is the seventh most leading cause of cancer deaths in the world, and the rate of incidence is parallel to the rate of mortality due to pancreatic cancer [193]. To evaluate the role of AKBA, different in vitro studies on pancreatic cancer cell lines, such as AsPC-1 and PANC-28, and in vivo studies were performed. AKBA was found to inhibitcell growth and downregulate the expressions of Ki-67, CD31, Cox-2, MMP-9, CXCR4, and VEGF in the tumor tissues [90]. Recently, combination of the anti-diabetic drug metformin and BA nanoparticles showed synergism in inhibiting the growth of pancreatic cancer cells [194].

#### 3.4.11. Melanoma

Mainly the population of the world bearing white skin is prone to melanoma, and it is considered as a serious global concern [195]. The effect of an isomeric compound, BC-4, containing both α- and β-BA acetate was studied via in vitro study. It was observed that BC-4 was responsible for the induction of B16F10 cells differentiation, blockage of the cell population in the G1 phase of the cell cycle, attenuation of topoisomerase II activity as well as the migratory potential of B16F10 cells when administered at a concentration of 25µM for 48h. Further, in fibrosarcoma cells, HT-1080 apoptosis was induced, and MMP secretion was reduced after treatment with BA [96].

### 3.5. Renal Intestinal Fibrosis

The role of AKBA in renal-intestinal fibrosis was studied both in vitro and in vivo using hypoxia-induced HK-2 cells and C57BL/6 mice, respectively via unilateral ureteral obstruction (UUO). The findings showed that AKBA exhibited a renoprotective effect via modulation of the Klotho/TGF-β/Smad signaling pathways. Hence, AKBA can be employed effectively for the treatment of renal-intestinal fibrosis [159].

### 3.6. Inflammatory Bowel Diseases (IBDs)

IBDs can be defined as idiopathic chronic relapsing malfunctions of the gastrointestinal tract (GIT) with an unknown origin, is characterized by the heterogeneity and multifactorial nature of their pathogenesis [196]. Ulcerative colitis affects the colon, where leukotrienes play a significant role. A study on effects of the BSE in patients with ulcerative colitis illustrated that administration of BSE for six weeks improved the stool properties, histopathology, and blood parameters, including Hb, serum iron, calcium, phosphorus, proteins, total leukocytes, and eosinophils [197]. Further, in an attempt to study the effect of AKBA on experimental ileitis, it was observed that treatment with AKBA caused a significant decrease in rolling (up to 90%) and adherent (up to 98%) leukocytes. Also, high doses of *Boswellia* extract, as well as AKBA, significantly reduced tissue injury scores [198]. Moreover, in an investigation on the effects of BSE in mouse models of chemically induced colitis, it was found that *BA* was incapable of ameliorating the symptoms of colitis and it exerted hepatotoxicity at higher doses [199]. Contrary to this report, another study demonstrated the anti-inflammatory effect of the semisynthetic form of AKBA and showed that P-selectin-regulated recruitment of inflammatory cells may be a major site of action for this novel anti-inflammatory agent in dextran sodium sulfate (DSS)-induced experimental murine colitis [200].

### 3.7. Diabetes

Diabetes is becoming the leading causes of death worldwide. It is classified into two types—Type1 diabetes (T1D) and Type 2diabetes(T2D).T1D is an autoimmune disorder whereas T2D is a metabolic disorder [201]. A study on alloxan-induced diabetic rats reported significant hypoglycemic effects on the continued use of the aqueous extract of leaves and roots of *Boswellia glabra*. Moreover, a decrease in the serum glucose level, cholesterol, triglyceride, urea and creatinine levels, and enzyme activities (alkaline phosphatase and glucose-6-phosphatase) was observed after treatment [144]. Also, it was observed that the administration of BSE can cause a significant decrease in blood glucose level along with HbA1c, cholesterol, LDL, and fructosamine [145,146,202]. Likewise, the isolated compounds from the plant, such as KBA and AKBA prevented the occurrence of autoimmune reactions, insulitis, and reduced hyperglycemia in multiple low-dose streptozotocin (MLD-STZ)-induced diabetes models [147].

### 3.8. Central Nervous System Disorders

BAs may also have tremendous potential in the treatment of central nervous system disorders such as Parkinson’s, Alzheimer’s disease, and cognitive impairment. Treatment with BAs has shown reduced inflammatory markers, improved general motor performance, nigral tyrosine hydroxylase immunostaining, and increased striatal dopamine levels in Parkinsonian rats [134]. The effects of α-BA were investigated in primary fetal human astrocytes under a stress paradigm as a probable model for Alzheimer’s disease. The results showed that α-BA could be considered as an effective remedy for prevention and lessening the progression of Alzheimer’s hallmarks in astrocytes; though, further preclinical findings are critical [109]. In a neuroinflammatory model of mice, AKBA showed antiapoptotic and anti-amyloidogenic effects via modulation of miRNA-155 [161]. Moreover, BA exhibited a neuroprotective role in Wistar rat models of cognitive impairment [203]. In another model of cognitive dysfunction, combination treatment with AKBA and celecoxib exhibited anti-inflammatory, antiglutamatergic, and antiamyloidogenic properties, leading to better prognosis of the disease [119].

### 3.9. Ischemia-Reperfusion Injury (IRI)

IRI is a physiopathological condition involving numerous metabolic processes which finally leads to cell apoptosis and ultimately tissue necrosis [204]. The protective effect of KBA against myocardial IRI in rats was observed. Three dose levels of KBA exerted dose-dependent cardioprotective effects, as manifested by a dose-dependent drop in serum lactate dehydrogenase and infarct size [149]. In ischemic brain injury also, AKBA was responsible for neuroprotection that involved the Nrf2/HO-1 defense pathway. It was found that the administration of AKBA increased Nrf2 and HO-1 expression, and a similar observation was also made for the compound KBA against cerebral ischemia-reperfusion injury [150,151].

### 3.10. Psoriasis

The gum resin of *B. serrata* has been also found effective in curing diverse skin problems such as psoriasis. A study was conducted to evaluate the effect of AKBA using murine bone marrow-derived dendritic cells (BMDCs) and a psoriasis-like mouse model, respectively. The results confirmed the anti-inflammatory effects of AKBA on psoriasis via modulation of IRF and TLR7/8 signaling pathways [140].

### 3.11. Other Diseases

Apart from the above mentioned diseases, a few reports on other diseases are also available where positive effects of BAs have been observed. In a study on guinea pigs with experimental autoimmune encephalomyelitis, BAs were found to reduce the clinical symptoms of the disease [205]. In an attempt to assess the antiulcer properties of BA, it was found to inhibit the ulcer formation in different experimental models. It was suggested that the protective action comes from enhanced gastric mucosal resistance, cytoprotective prostaglandins synthesis, and leukotriene synthesis inhibition [154]. A study on the gastroprotective role of α-BA was performed in ethanol-induced gastric injury in rats. The findings demonstrated that α-BA decreased ethanol administration related injuries, gastric juice acidity, and the development of MDA, and improved CAT activity along with SOD activity and the level of NO and PGE-2 [153]. In the case of myocardial injury, AKBA in combination with HSYA showed cardioprotective effects via modulation of the PGC-1α/Nrf2 pathway [132]. In another study, the efficacy ofBA against acetaminophen (APAP)-induced hepatotoxicity in Balb/c mice was determined. It was observed that BA pre-intake reduced APAP-induced production of inflammatory cytokines and chemokines. Further, it affected the expression of NF-κB p65 and p-JNK, TLR-3, TLR-4, and MyD88 [155,206].

Recently, BSE in combination therapy with curcumin was found to inhibit chikungunya and vesicular stomatitis virus infections *in vitro*. The combination therapy was able to block the entry of CHIKV Env-pseudotyped lentiviral vectors, and they suppressed CHIKV infection *in vitro*. Furthermore, vesicular stomatitis virus vector particles and viral infections were also reduced, thereby demonstrating its broad antiviral activity [143]. An in vitro study was performed to explore the ethnomedicinal use of BSE, and it was proved that BSE, as well as BA, efficiently inhibited wild-type and a clinical isolate of HSV-1 via alteration of NF-κB and p38 MAPK pathways [157]. Furthermore, in another study on a mouse model of LPS-induced neuroinflammation, AKBA played a significant role in counteracting the symptoms via modulation of miRNA-155 [161]. Treatment of mouse models of Ehrlich tumor and Ehrlich ascites carcinoma with BA have demonstrated the antitumor property of the compound by interfering with the IL-6-STAT-3 signal transduction pathway. APOBA treatment on Ehrlich ascites carcinoma (EAC) cells and sarcoma 180 (S-180) cells also witnessed tumor growth inhibition [115,120,121]. In the pulmonary arterial hypertensive rat model, α-BA administration showed protective effects by downregulating the expression of JNK and protein kinase 1 under hypoxic conditions [142]. IN0523 (Urs-12-ene-3α,24β-diol), a derivative of BA, was found to show protective effects in response to cisplatin-induced urogenital toxicity by inhibiting the imbalance of oxidative stress/redox state and by enhancing the efflux mechanisms [160].

## 4. Boswellic Acid Implicated in Different Phases of Human Clinical Trials

As aforementioned, not only the preclinical studies but also the studies carried out in the clinical settings well evinced the high potential of BA against diverse chronic diseases(Table 2). In a double-blind, placebo-controlled human trial, oral administration of Boswellin, a formulation containing AKBA and BBA, was found to exert anti-inflammatory/antiarthritic effects in osteoarthritis patients [207]. Further, a novel BSE containing 30% AKBA and known as 5-Loxin led to improved physical functioning and decreased pain in patients with osteoarthritis, plausibly via regulation of inflammatory responses by decreasing pro-inflammatory modulators and enzymatic degradation of cartilage, without exerting any toxic effect [208]. Also, a comparative, randomized, double-blind, placebo-controlled study, which examined the efficacy and safety of curcumin in combination with BA, exhibited favorable responses in patients with osteoarthritis [209]. Additionally, another combination of BA with methylsulfonylmethane (MSM) also displayed satisfactory outcomes in the treatment of knee arthritis [210]. However, Notarnicolaet al. showed this combination not to exert much efficacy in the case of gonarthrosis [211]. Additionally, a lecithin-based delivery form of *B.serrata,* named as Casperome^®^, was reported to improve the signs and symptoms of patients with irritable bowel syndrome in a highly safe and effective fashion [212]. A double-blind placebo-controlled study as well witnessed that administration of BSE containing BAs in major proportions led to significant improvement in patients suffering from bronchial asthma. The symptoms such as dyspnoea, rhonchi, number of attacks, increase in FEV subset1, FVC, and PEFR, in addition to a decline in the eosinophilic count and ESR, were evidenced [213]. Further, treatment with BSEdecreased cerebral edema in patients irradiated for brain tumors significantly, as evinced by a prospective, randomized, placebo-controlled, double-blind pilot trial [214]. Besides, a clinical trial conducted by Gerhardt and group to compare the efficacy and safety of H15, a BSE with mesalazine for the treatment of active Crohn’s disease, H15 was found to exert better effect concerning a benefit–risk evaluation [215]. Also, In a double-blind study, it was witnessed that a novel BA formulation (consisting of Bosexil(^®^), INCI (International Nomenclature of Cosmetic Ingredients): lecithin, an extract of *B. serrata* resin) was a favorable candidate for therapy of patients suffering from erythematous eczema and psoriasis [216]. Furthermore, the topical application of a cream containing 0.5% BA presented a well–tolerated and safe treatment approach for photoaged skin [217,218]. Thus, these studies clearly indicate BA to be safe, well-tolerated, and effective, and thus implies its high therapeutic potential against a wide array of human chronic diseases.

## 5. Pharmacokinetic Properties of Boswellic Acids

BAs are the chief bioactive element of frankincense, and various studies have established their bioactivities. However, to develop it as a successful candidate drug, its pharmacokinetic properties must be considered accurately. In this regard, the studies conducted have exhibited poor pharmacological performance. Both KBA and AKBA are extremely lipophilic drugs, which result in reduced absorption through the GIT, but they also exhibit high retention time. Preliminary pharmacokinetic studies have shown minimal concentrations of both AKBA and KBA in human plasma after administration of BSE [41,219,220,221,222,223]. Moreover, the incidence of AKBA in plasma is uncertain due to its deacetylation to KBA in vivo [222]. Contrary to this report, another study on the metabolism of BAs indicated that AKBA is not deacetylated to KBA. Furthermore, it was demonstrated that unlike AKBA, KBA experiences extensive phase I metabolism in rat and human liver microsomes, as well as in hepatocytes. The metabolic profiles of KBA in rat plasma and liver were found to be similar in both in vitro and in vivo study whereas no metabolites of AKBA could be recognized. This indicates that the administration step should be further implemented to increase the bioavailability of AKBA [224]. Another study reported that the foremost permeability-associated barriers that compromised oral bioavailability of KBA include its gastrointestinal volatility, CYP3A4, mediated intestinal metabolism, accumulation within the enterocytes, and saturable kinetics [225]. In another study, the metabolic stability, permeability and brain availability of six major BAs, i.e., KBA, AKBA, βBA, 3-acetyl-β-BA (AβBA), αBA, and 3-acetyl-α-BA (AαBA) was evaluated. The four BAs lacking the 11-keto moiety showed reasonable permeability. In contrast to AαBA and AβBA, βBA, and αBA were effectively metabolized and also the availability of all six major BAs was confirmed in rat brain eight hours after oral administration of 240 mg/kg BSE to rats [226].

One more important pharmacokinetic parameter to be kept in mind while experimenting with a drug is its elimination from the body. A study reported that the elimination half-life of BA is about six hours. This suggests that oral administration of the medication is required after every six hours. The study also reported that a stable state of the drug would be achieved in the plasma after around thirty hours [222].

Several studies have been designed to analyze the biotransformation of BAs within the body. One such study explored the different probable derivatives of BA via biotransformation by Cunninghamella blakesleana AS 3.970. As many as ten transformed compounds, including 7β-hydroxy-11-keto-β-boswellic acid; 7β, 15α-dihydroxy-11-keto-β-boswellic acid; 7β, 16β-dihydroxy-11-keto-β-boswellic acid; 7β, 16α-dihydroxy-11-keto-β-boswellic acid; 7β, 22β-dihydroxy-11-keto-β-boswellic acid; 7β, 21β-dihydroxy-11-keto-β-boswellic acid; 7β, 20β-dihydroxy-11-keto-β-boswellic acid; 7β, 30-dihydroxy-11-keto-β-boswellic acid; 3α, 7β-dihydroxy-11-oxours-12-ene-24; 30-dioic acid; and 3α, 7β-dihydroxy-30-(2-hydroxypropanoyloxy)-11-oxours-12-en-24-oic acid were extracted and purified through hydroxylation, oxidation, and esterification, and their chemical structures were characterized by various spectroscopic methods [227].

## 6. Improvement in the Bioavailability of Boswellic Acids

In regard of the relatively low plasma and brain levels of BAs, and as a consequence of their inability to inhibit 5-LOX in whole blood, the abrogation of LTB4 synthesis in vivo by frankincense extracts remains unclear. For exploiting the potential pharmacological properties of different BAs, several approaches have been used to enhance its bioavailability [228]. Some researchers have also tried to enhance the bioavailability of BAs by administering it with a standardized meal [229]. Also, an improvement in their uptake was observed when it was administered with anionic drugs [230]. Further, different methods such as lecithin delivery form (PhytosomeR); nanoparticle delivery systems like liposomes, emulsions, solid lipid nanoparticles, nanostructured lipid carriers, micelles, and poly (lactic-co-glycolic acid) nanoparticles; and synthetic derivatization of BA have been adapted for overcoming these limitations [231,232,233]. Formulation of BA with lecithin was found to improve absorption and tissue penetration of BA in a single-dose, randomized, open-label study [234].

## 7. Conclusions

BAs, the pentacyclic triterpenic acids comprising of α-,β-,γ-BA,acetyl-β-BA, KBA, AKBA, and so on, have exhibited diverse pharmacological activities against various chronic diseases, as evidenced through the multiple preclinical studies and various clinical trials. They can target several key players involved in the pathogenesis of these diseases. It was observed that different important molecular targets are affected by BA treatment, such as LO, MAPK, NF-κB, TNF-α, Erk-1/2, etc., which plays an imperative role in the development of various chronic diseases. Yet, concerns regarding the pharmacokinetic properties have had major dampening effects in the path of development of this compound as an effective drug. Nevertheless, many investigations have been initiated in this matter to triumph over the limitations, but the pace is quite slow, and an ample amount of attention is needed.

## Figures and Tables

**Table 1:** Biological activity of boswellic acid against different diseases.

Diseases	Mechanism/Outcome	References
Arthritis	↓ Infiltration of leucocytes	[105]
	↓ Knee diameter	[106]
	↓ IL-1β and TLR4, ↑ Synovial activation	[107]
RA-derived bone loss disease	↓TNF-α and NF-κB activity	[108]
Alzheimer’s disease	↑ Reeling expression, ↓ ROS generation	[109]
Asthma	↓ Expression of pSTAT6 and GATA3	[110]
	↓ Expression of pSTAT6 and GATA3	[111]
Atherosclerosis	↓ NF-κB activity	[86]
Breast cancer	↑ ER/UPR response	[112]
Bladder cancer	↑ Tumor cell specific cytotoxicity	[113]
Brain cancer	↓ Phosphorylation of Erk-1 and Erk-2	[84]
	↑ Apoptosis	[114]
Cervical cancer	↑ PARP cleavage	[115]
Colon cancer	↑ let-7, CDK6, vimentin, and E-cadherin	[34]
	↓ 4E and cyclin D1, ↓ G2/M cell cycle	[42]
	↓ Intestinal tumorigenesis	[91]
	↓ Cyclin D1 and E, CDK 2 and 4	[87]
	↑ PARP cleavage	[115]
	↓ Caspase-3 or caspase-8	[116]
	↑ Expression of SAMD14 and SMPD3	[117]
	↑ Apoptosis	[118]
Cognitive impairment	↓ Glutamate level	[119]
Ehrlich tumor	↓ NF-κB and tumor growth,↑ PARP cleavage	[93]
	↑ PARP cleavage and apoptosis	[115]
	↑ Tumor cell apoptosis	[120]
	↑ Caspase-3, and apoptosis	[121]
Glioma	↑ p21 via p53-independent pathway	[122]
	↓ Growth of C6 glioma	[123]
	↓ Topoisomerase I	[95]
	↑ Apoptosis	[114]
	↓ Topoisomerases I and II	[97]
Glioblastoma	↓ G2/M phase, p21/FOXM1/cyclin B1	[124]
	↓ p53 and Bcl-2, ↓ IĸB-α	[125]
Myeloid Leukemia	↑ Apoptosis	[126]
	↑ Caspase-3 and -8, and DR4 and DR5	[127]
	↓ PI3K/Akt/Hsp-90 cascade	[128]
	↓ DNA synthesis	[129]
Liver cancer	↑ Caspase-3 and -8 dependent apoptotic pathway	[130]
Lung cancer	↓ NF-κB signaling	[89]
	↑ Apoptosis	[123]
	↑ PARP cleavage, apoptosis	[115]
	↑ PARP cleavage, JNK pathway	[131]
Melanoma	↓ Topoisomerase II, and MMPs	[96]
Meningioma	↓ Phosphorylation of Erk-1 and Erk-2	[84]
Myocardial injury	↓ CK-MB and LDH	[132]
Neuroblastoma	↑ PARP cleavage, ↑ Apoptosis	[115]
Pancreatic cancer	↓ COX-2, MMP-9, CXCR4, and VEGF	[90]
	↓ p-mTOR, p-p70S6K (T389), p-4EBP and p-S6	[133]
Parkinson’s disease	↓ Inflammatory markers	[134]
Prostate cancer	↓ NF-κB signaling, Bcl-2, and Bcl-x(L)	[88]
	↑ DR5-mediated pathway	[99]
	↓ AR signaling, ↑ p21(WAF1/CIP1)	[100]
	↓ Tumor growth and angiogenesis	[101]
	↑ Caspase 3 and apoptosis	[135]
	↓ mTOR signaling	[136]
	↑ PARP-1 cleavage, ↓ tumor growth	[137]
	↓ Akt and STAT3 signaling	[138]
	↓ Cyclin D1, and Pin1	[139]
Psoriasis	↓ IL-12, IL-23, TLR7/8, and IRF	[140]
	↓ SAM/SAH ratio	[141]
Pulmonary arterial hypertension	↓ Apoptosis and proliferation	[142]
Chikungunya	↓ Entry of CHIKV Env-pseudotyped lentiviral vectors	[143]
Diabetes	↑ Synthesis of secretory granules	[144]
	↓ Islet destruction and consequent hyperglycemia	[145]
	↑ Blood glucose and HbA1c	[146]
	↓ Cytokine burst, and blood glucose	[147]
	↓ Infiltration of lymphocytes into pancreatic islets	[148]
Ischemia-reperfusion	↑ Antioxidant capacity, ↓ inflammatory cascades	[149]
	↑ Nrf2 and HO-1	[150]
	↑ Nrf2 and HO-1	[151]
	↓ Brain infarction, neuronal cell loss, and apoptosis	[152]
Gastric injury	↑ Nrf2 and HO-1	[153]
Gastric ulcer	↓ Biosynthesis of leukotrienes	[154]
Hepatic injury	↓ Glutathione, and ROS	[155]
Hepatotoxicity	↑ Nrf2 and HO-1	[156]
HSV-1 infection	↓ NF-κB, p38 MAP-kinase, TNF-α,IL-1β, and IL-6	[157]
Ileocecal adenocarcinoma	↑ Rhodamine (Rh123), ↓P-gp, andMDR gene1	[158]
Renal intestinal fibrosis	↓ TGFβ-RI, TGFβ-RII, p-Smad2/3, and Smad4	[159]
Urogenital toxicity	↓ Glutathione peroxidase, catalase, and SOD	[160]
Neuroinflammation	↓ P-IκB-α, miRNA-155 expression level	[161]

**Abbreviations:** IL-1β= interleukin 1beta; TLR4= toll-like receptor 4; RA= rheumatoid arthritis; TNF-α=tumor necrosis factor α; NF-κB= nuclear factor kappa-light-chain-enhancer of activated B cells; ROS= reactive oxygen species;pSTAT6= phospho-signal transducer and activator of transcription 6; ER/UPR= endoplasmic reticulum/unfolded protein response; Erk= extracellular-signal-regulated kinase; PARP= poly-ADP ribose polymerase; let-7= lethal-7; CDK6=cyclin-dependent kinase 6; FOXM1= the forkhead box m1; Bcl-2= B-cell lymphoma2; PI3K= phosphoinositide 3-kinase; Hsp-90= heat shock protein90; AOM= acute otitis media; JNK= c-Jun N-terminal kinase; MMPs= matrix metalloproteinase; Erk= extracellular signal-regulated kinase; CK-MB= creatine kinase-muscle/brain; LDH= lactate dehydrogenase; COX-2= cyclooxygenase-2; CXCR4= C-X-C motif chemokine receptor 4; VEGF= vascular endothelial growth factor; Mtor = mammalian target ofrapamycin; p70S6K= P70 S6 kinase; IRF= impulse response function; SAM= S-adenosylmethionine; CHIKV= chikungunyavirus; HbA1c= hemoglobin A1c; Nrf2= nuclear factor erythroid 2-related factor 2; HO-1= heme oxygenase-1; MAPK= mitogen-activated protein kinase; Rh123= rhodamine 123; P-gp=P-glycoprotein1; MDR= multidrug-resistant; TGFβ-R= transforming growth factor beta receptor.

**Table 2:** Application of boswellic acid in different phases of human clinical trials.

Disease	Dosage/Clinical Outcomes	References
Osteoarthritis^a,B^	(500 mg)/↓pain-related symptoms*	[209]
Osteoarthritis^b,C^	(100, 250 mg)/↓pain and ↑ physical functioning*	[208]
Osteoarthritis^C^	(300-500 mg)/↓pain and stiffness*	[207]
Knee arthritis^c,C^	(7.2 mg)/good and satisfactoryeffect*	[210]
Gonarthrosis^c,C^	(7.2 mg)/highly effective*	[211]
Brain tumors^A^	(4200 mg)/↓ cerebral edema*	[214]
Photoaged skin^C^	(0.5 %)/well-tolerated withoutadverse effects*	[217]
Crohn disease^d,C^	(NIL)/well tolerated*	[215]
Diabetes^C^	(NIL)/↑ blood HDL levels, and ↓cholesterol*	[202]
Erythematous eczema^C^	(NIL)/improvement in symptoms*	[216]
Asthma^C^	(300 mg)/↓eosinophilic count and ESR*	[213]

**Abbreviations:**^a^= BA in combination with curcumin; ^b^ = 5-Loxin, a novel Boswellia serrata extract enriched with 30% AKBA; ^c^ = BA in combination with methylsulfonylmethane; ^d^= Boswellia serrata extract H15;^A^= Phase I; ^B^ = Phase II; ^C^ = NA, HDL = high density lipoprotein; ESR= erythrocyte sedimentation rate.*= All the studies listed above are completed.
